# LCAO Electronic Structure of Nucleic Acid Bases and Other Heterocycles and Transfer Integrals in B-DNA, Including Structural Variability

**DOI:** 10.3390/ma14174930

**Published:** 2021-08-30

**Authors:** Marilena Mantela, Constantinos Simserides, Rosa Di Felice

**Affiliations:** 1 Department of Physics, National and Kapodistrian University of Athens, Panepistimiopolis, Zografos, GR-15784 Athens, Greece; mmantela@phys.uoa.gr; 2 Department of Physics and Astronomy and Department of Quantitative and Computational Biology, University of Southern California, Los Angeles, CA 90089, USA; 3 CNR-NANO Modena, I-41125 Modena, Italy

**Keywords:** charge transfer, DNA, nucleic acids, Linear Combination of Atomic Orbitals (LCAO), Molecular Dynamics (MD), Tight Binding (TB), heterocycles

## Abstract

To describe the molecular electronic structure of nucleic acid bases and other heterocycles, we employ the Linear Combination of Atomic Orbitals (LCAO) method, considering the molecular wave function as a linear combination of all valence orbitals, i.e., 2s, 2p${}_{x}$, 2p${}_{y}$, 2p${}_{z}$ orbitals for C, N, and O atoms and 1s orbital for H atoms. Regarding the diagonal matrix elements (also known as on-site energies), we introduce a novel parameterization. For the non-diagonal matrix elements referring to neighboring atoms, we employ the Slater–Koster two-center interaction transfer integrals. We use Harrison-type expressions with factors slightly modified relative to the original. We compare our LCAO predictions for the ionization and excitation energies of heterocycles with those obtained from Ionization Potential Equation of Motion Coupled Cluster with Singles and Doubles (IP-EOMCCSD)/aug-cc-pVDZ level of theory and Completely Normalized Equation of Motion Coupled Cluster with Singles, Doubles, and non-iterative Triples (CR-EOMCCSD(T))/aug-cc-pVDZ level of theory, respectively, (vertical values), as well as with available experimental data. Similarly, we calculate the transfer integrals between subsequent base pairs, to be used for a Tight-Binding (TB) wire model description of charge transfer and transport along *ideal* or *deformed* B-DNA. Taking into account all valence orbitals, we are in the position to treat deflection from the planar geometry, e.g., DNA structural variability, a task impossible for the plane Hückel approach (i.e., using only 2p${}_{z}$ orbitals). We show the effects of structural deformations utilizing a 20mer evolved by Molecular Dynamics.

## 1. Introduction

The study of the electronic structure of organic heterocyclic molecules has been of interest for the scientific community for decades, especially since the establishment of investigation methods based on quantum mechanics. This includes the electronic structure and properties of nucleic acid oligomers and polymers, DNA and RNA. The sequence of bases, adenine (A), thymine (T) or uracil (U), guanine (G), cytosine (C), is where genetic information is stored and transferred in all living organisms. The understanding of its electronic structure and charge transfer [1] properties is a crucial issue in biology, involved in functions such as damage and repair, carcinogenesis and mutagenesis [2,3,4], mutations and diseases [5,6,7,8] and is also important for novel applications in nanotechnology [9,10].

The last two decades have witnessed a surge of studies of DNA as the basis for molecular wires and molecular electronics devices/circuits, based on self-assembly and specific base hybridization [11,12,13,14,15]. The prospect of using DNA in materials science stems from exploiting its properties of molecular recognition, assembly, and processing information [11] as well as its ability to transfer or transport charge. Among other theoretical and experimental attempts, the electronic structure of single DNA molecules has been resolved by transverse scanning tunneling spectroscopy and assigned to groups of orbitals originating from the molecular entities, i.e., nucleobases, backbone, counterions [12]. Properties of long-range charge transport in DNA and DNA-mediated charge transfer and mechanisms have been studied a for a long time now [13]. Furthermore, currents in the range of 10–100 pA have been measured in G4-DNA over distances in the range of 10–100 nm [14]. Today, DNA plays an increasingly important role in molecular electronics due to its structural and molecular recognition properties [15].

In this work, we calculate the ionization and excitation energies of nucleic acid bases and similar molecules as well as assemblies of DNA bases using a semi-empirical Linear Combination of Atomic Orbitals (LCAO) method that includes all valence orbitals with a novel parameterization developed by us. Additionally, using this approach, we obtain electronic parameters for charge (electron or hole) transfer along DNA, which can be employed to model electron and hole conductivity. We investigate the electronic structure of the four DNA bases A, T, G, C and of the two Watson–Crick H-bonded pairs A-T and G-C. We focus on the HOMO (Highest Occupied Molecular Orbital) and LUMO (Lowest Unoccupied Molecular Orbital) wave functions and energies. With the new LCAO parameterization developed by us in this work, we calculate the transfer matrix elements between stacking base pairs, for all possible combinations between them, for both electrons and holes, aiming at parameterizing a Tight-Binding (TB) wire model. We calculate the transfer matrix elements for *ideal* geometries, namely for planar bases and base pairs separated and twisted approximately by 3.4 Å and 36${}^{\circ }$, respectively, relative to the double helix growth axis. Our results are compared with published experimental and computational (from first principles and simpler TB models) data for the HOMO and LUMO energies. Finally, the deformed base pairs pruned from several snapshots of a 500 ns Molecular Dynamics (MD) trajectory of a 20mer [16] are used in order to address the effects of structural variability in the electronic structure and charge transfer properties of B-DNA within the LCAO approach.

The rest of this article is organized in the following way: In Section 2, we develop the novel LCAO parameterization that includes all valence orbitals for nucleic acid bases (Section 2.1) and base pairs (Section 2.2). This methodology is not limited to these specific molecular systems but can be applied to similar heterocycles. Next, we obtain the TB parameters that are relevant for a wire model description of charge transfer and transport along B-DNA (Section 2.3). We also describe non ideal bases and base pairs obtained by MD (Section 2.4). In Section 3, we present our results on ionization and excitation energies of various heterocyclic planar molecules, including isolated DNA bases (Section 3.1). The on-site energies of base pairs and transfer integrals between stacked base pairs are presented in Section 3.2. We study the effects of structural variability on the electronic structure and charge transfer properties of B-DNA in Section 3.3. Finally, Section 4, contains our overall conclusions.

## 2. Theory

### 2.1. LCAO with All Valence Orbitals for Nucleic Acid Bases or Similar Molecules

We consider the state │β〉 of a nucleic acid base, or a similar molecule, as a linear combination of all valence orbital states ${│\phi }_{i\nu }\rangle$, i.e., 2s, 2p${}_{x}$, 2p${}_{y}$, 2p${}_{z}$ for C, N, and O atoms, and 1s for H atoms:

(1) $$│\beta \rangle =\sum_{\nu =1}^{N}\sum_{i=1}^{I}c_{i\nu }{│\phi }_{i\nu }\rangle .$$

The index ν runs among all *N* atoms of the molecule and the index *i* runs among all *I* orbital states of each atom, respectively. │β〉 obeys the Schrödinger equation

(2) $${\hat{H}}_{B}│\beta \rangle =E_{B}│\beta \rangle .$$

${\hat{H}}_{B}$ is the Hamiltonian of the base (or other molecule), with eigenvalues $E_{B,k}$ and eigenvectors ${│\beta \rangle }_{k}$. Taking the bracket, using ${\langle \phi }_{j\mu }│$, Equation (2) gives the linear system of equations

(3) $$\sum_{\nu =1}^{N}\sum_{i=1}^{I}\left[\left(H_{B,j\mu i\nu }-E_{B}\;S_{j\mu i\nu }\right)c_{i\nu }\right]=0,\;\mu =1,\cdots ,N,\;j=1,\cdots ,I.$$

The Hamiltonian matrix elements $H_{B,j\mu i\nu }$ are given by

(4) $$H_{B,j\mu i\nu }=\langle \phi_{j\mu }│{\hat{H}}_{B}{│\phi }_{i\nu }\rangle$$

and the overlap matrix elements are

(5) $$S_{j\mu i\nu }=\langle \phi_{j\mu }{│\phi }_{i\nu }\rangle \approx \delta_{j\mu i\nu }.$$

We notice that we have approximated $S_{j\mu i\nu }$ by $\delta_{j\mu i\nu }$. The system of Equation (3) is solved by numerical diagonalisation, giving the eigenenergies $E_{Bk}$ and eigenvectors

(6) $$│\beta_{k}\rangle =\left[\begin{array}{c} c_{11k}\\ c_{12k}\\ \vdots \\ c_{i\nu k}\\ \vdots \\ c_{INk} \end{array}\right].$$

To this end we need the values of the Hamiltonian matrix elements, $H_{B,j\mu i\nu }$. Regarding the diagonal matrix elements $H_{B,i\nu i\nu }$—also known as on-site energies—we utilize a novel parameterization, namely: $E_{H(1s)}=-13.64$ eV for H 1s orbitals, $E_{C(2s)}=-13.18$ eV for C 2s orbitals, $E_{C(2p)}=-6.70$ eV for C 2p orbitals, $E_{N(2s)}=-14.51$ eV for N 2s orbitals, $E_{N(2p)}=-9.55$ eV for N 2p orbitals, $E_{O(2s)}=-15.03$ eV for O 2s orbitals, $E_{O(2p)}=-11.52$ eV for O 2p orbitals. As for the nondiagonal matrix elements $H_{B,j\mu i\nu }\;\left(\mu \neq \nu \right)$ referring to neighboring atoms, we utilize the Slater–Koster two-center interaction transfer integrals [17]

(7) $$\begin{array}{ccc} V_{\mathrm{ss}} & = & V_{\mathrm{ss}\sigma }, \end{array}$$

(8) $$\begin{array}{ccc} V_{\mathrm{sx}} & = & \xi_{1}\;V_{\mathrm{sp}\sigma }, \end{array}$$

(9) $$\begin{array}{ccc} V_{\mathrm{xx}} & = & \xi_{1}^{2}\;V_{\mathrm{pp}\sigma }+\left(1-\xi_{1}^{2}\right)\;V_{\mathrm{pp}\pi }, \end{array}$$

(10) $$\begin{array}{ccc} V_{\mathrm{xy}} & = & \xi_{1}\xi_{2}\;\left(V_{\mathrm{pp}\sigma }-V_{\mathrm{pp}\pi }\right), \end{array}$$

with $\xi_{1}$, $\xi_{2}$ being the directional cosines of $\vec{d}=\vec{ji}$ which points from atom *i* to atom *j*. Concerning the values of $V_{\mathrm{ss}\sigma },V_{\mathrm{sp}\sigma },V_{\mathrm{pp}\sigma }$, $V_{\mathrm{pp}\pi }$, we use the relevant expressions proposed by Harrison [18,19], of the form:

(11) $$V_{\chi }=\chi \frac{\hbar^{2}}{md^{2}},$$

with *m* being the electron mass and *d* being the two-center distance. The χ values that we propose here are: $\chi_{\mathrm{ss}\sigma }=-1.32$, $\chi_{\mathrm{sp}\sigma }=-1.42$, $\chi_{\mathrm{pp}\pi }=-0.73$ (slightly modified relative to the original Harrison constant), $\chi_{\mathrm{pp}\sigma }=2.22$. For each H orbital, the interactions are multiplied by a factor b=0.70 that resulted from the optimization. We arrived at the above parameterization after careful optimization by fitting the LCAO numerical results with the experimental values for the excitation and the ionization energies of nucleic acid bases A, G, T, C, and U. To do so, we used the Nelder–Mead algorithm as implemented in Matlab software. All other nondiagonal matrix elements, referring to non-neighboring atoms, are assumed equal to zero, $H_{B,j\mu i\nu }=0$. In Table 1 and Table 2 we summarize our LCAO parameters.

From the numerical diagonalization of the Hamiltonian matrix, one obtains the energy eigenvalues corresponding to the electronic spectrum of molecular orbitals. The occupied and unoccupied orbitals—and thus the HOMO and LUMO—can be found by counting all valence electrons contributed by the atoms of the molecule and arranging them successively in couples of different spin in accordance with the Pauli principle. The same treatment developed for DNA bases is applicable to other purines, pyrimidines, and similar molecules.

### 2.2. LCAO with All Valence Orbitals for B-DNA Base Pairs

Likewise, we obtain the HOMO and LUMO states of a B-DNA base pair or *monomer*. Let us call $N_{1}$, $N_{2}$ the number of atoms making up the two bases of the base pair. We consider the base pair or monomer state │α〉 as a linear combination of all valence orbital states ${│\phi }_{i\nu }\rangle$, i.e., 2s, 2p${}_{x}$, 2p${}_{y}$, 2p${}_{z}$ for C, N and O atoms and 1s for H atoms:

(12) $$│\alpha \rangle =\sum_{\nu =1}^{N_{1}+N_{2}}\sum_{i=1}^{I}c_{i\nu }{│\phi }_{i\nu }\rangle .$$

The indexes ν and *i* run among the $N_{1}+N_{2}$ atoms of the base pair and the *I* orbitals of each atom, respectively. │α〉 obeys the Schrödinger Equation

(13) $${\hat{H}}_{A}│\alpha \rangle =E_{A}│\alpha \rangle .$$

│α〉 and $E_{A}$ are the eigenvectors and eigenenergies of the monomer or base pair Hamiltonian ${\hat{H}}_{A}$. By taking the bracket, using ${\langle \phi }_{j\mu }│$, Equation (13) gives the linear system of equations

(14) $$\sum_{\nu =1}^{N_{1}+N_{2}}\sum_{i=1}^{I}\left[\left(H_{A,j\mu i\nu }-E_{A}\;S_{j\mu i\nu }\right)c_{i\nu }\right]=0,\;\mu =1,\cdots ,N_{1}+N_{2},\;j=1,\cdots ,I.$$

The system of Equation (14) is solved by numerical diagonalisation, as well, giving the eigenenergies $E_{Ak}$ and eigenvectors

(15) $$│\alpha_{k}\rangle =\left[\begin{array}{c} c_{\alpha 1k}\\ c_{12k}\\ \vdots \\ c_{i\nu k}\\ \vdots \\ c_{I(N_{1}+N_{2})k} \end{array}\right].$$

In this case, the values of the Hamiltonian matrix elements, $H_{A,j\mu i\nu }$, are expressed slightly differently. The matrix elements $H_{A,j\mu i\nu }$ with (a) $1\leq \nu \leq N_{1}$ and $1\leq \mu \leq N_{1}$, and (b) $N_{1}+1\leq \nu \leq N_{1}+N_{2}$ and $N_{1}+1\leq \mu \leq N_{1}+N_{2}$, are expressed in the same way as previously described for molecules. For the remaining matrix elements, we employ the Slater–Koster two-center interaction transfer integrals of Equations (7), (8), (9), (10) but in this case, the values of $V_{ss\sigma },V_{sp\sigma },V_{pp\sigma },V_{pp\pi }$ are of the form

(16) $$V_{\chi }=\chi \;\frac{\hbar^{2}}{md_{0}^{2}}\;e^{-\frac{2}{d_{0}}\left(d-d_{0}\right)},$$

where $d_{0}=1.35$ Å is a typical covalent bond distance within a base. This difference stems from the fact that Harrison’s relations are valid for interatomic distances of the size of covalent bonds. However, the B-DNA bases (A and T, or G and C) are connected with noncovalent hydrogen bonds to form a base pair. The length of hydrogen bonds is longer than the typical length $d_{0}$ of the covalent bond connecting neighboring atoms within a base. Thus, when dealing with interatomic distances of the size of hydrogen bonds and longer, Harrison’s expressions of Equation (11) are replaced with the appropriate exponentially decaying expressions of the form of Equation (16) [20,21,22].

From the aforementioned diagonalization of the Hamiltonian matrix, we obtain the energy eigenvalues $E_{A}$—including HOMO and LUMO—of the electronic spectrum, as well as the corresponding eigenvectors (coefficients) $c_{i\nu }$ of a base pair.

### 2.3. Coherent Charge Transfer and Transport Parameters for a TB Wire Model

#### 2.3.1. Eigenstates

The HOMO or LUMO state of a DNA segment, made up of N monomers, can be expressed as

(17) $$│\mathrm{DNA}\rangle =\sum_{\alpha =1}^{N}v_{\alpha }│\alpha \rangle .$$

│α〉 is the HOMO or LUMO state of monomer (base pair) α and $v_{\alpha }$ are time-independent quantities. The Hamiltonian, in second quantization notation, in this TB wire model approach, can be written as

(18) $${\hat{H}}_{\mathrm{DNA}}=\sum_{\alpha =1}^{N}E_{\alpha }│\alpha \rangle \mathrm{\langle \alpha │}+\sum_{\alpha =1}^{N-1}t_{\alpha ,\alpha +1}│\alpha \rangle \langle \alpha +1│+\sum_{\alpha =2}^{N}t_{\alpha ,\alpha -1}│\alpha \rangle \langle \alpha -1│.$$

$E_{\alpha }$ is the HOMO or LUMO on-site energy of monomer α, and $t_{\alpha ,\gamma }$ is the transfer integral between monomers α and γ. By substituting Equations (17) and (18) into the time-independent Schrödinger equation

(19) $${\hat{H}}_{\mathrm{DNA}}│\mathrm{DNA}\rangle =E_{\mathrm{DNA}}│\mathrm{DNA}\rangle ,$$

we arrive to a system of N coupled equations

(20) $$E_{\alpha }v_{\alpha }+t_{\alpha ,\alpha +1}v_{\alpha +1}+t_{\alpha ,\alpha -1}v_{\alpha -1}=E_{\mathrm{DNA}}v_{\alpha }.$$

Equation (20) is equivalent to the eigenvalue-eigenvector problem

(21) $$H_{\mathrm{DNA}}\vec{v}=E_{DNA}\vec{v}.$$

$H_{\mathrm{DNA}}$ is the Hamiltonian matrix of order N composed of the TB parameters (on-site energies and transfer integrals) and $\vec{v}$ is the vector matrix composed of the coefficients $v_{j}$. The diagonalization of $H_{\mathrm{DNA}}$ leads to the determination of the HOMO or LUMO eigenenergy spectra (*eigenspectra*), $\left\{E_{k}\right\}$, k=1,2,⋯,N and of the occupation probabilities for each eigenstate, $|v_{jk}{|}^{2}$, where $v_{jk}$ is the *j*-th component of the *k*-th eigenvector.

#### 2.3.2. Coherent Charge Transfer

To describe charge transfer between stacked base pairs of double-stranded DNA, we suppose that an extra inserted electron travels through LUMOs, while an extra inserted hole travels through HOMOs. The time-dependent HOMO or LUMO state of the whole B-DNA segment, │DNA(t)〉, is considered as a linear combination of base-pair HOMO or LUMO states with time-dependent coefficients

(22) $$│\mathrm{DNA}(t)\rangle =\sum_{\alpha }A_{\alpha }(t)│\alpha \rangle ,$$

where │α〉 is the HOMO or LUMO state of the α-th monomer and the sum is extended over all monomers of the B-DNA segment. Substituting Equations (18) and (22) to the time-dependent Schrödinger equation

(23) $$i\hbar \frac{d│\mathrm{DNA}(t)\rangle }{dt}={\hat{H}}_{\mathrm{DNA}}│\mathrm{DNA}(t)\rangle ,$$

we obtain the system of N coupled differential equations:

(24) $$i\hbar \frac{A_{\alpha }}{dt}=E_{\alpha }A_{\alpha }+t_{\alpha ,\alpha -1}A_{\alpha -1}+t_{\alpha ,\alpha +1}A_{\alpha +1}.$$

Equation (24) is equivalent to a first-order matrix differential equation, which can be solved with the eigenvalue method.

#### 2.3.3. Coherent Charge Transport

To handle coherent charge transport in a TB approach, we also need the TB parameters (on-site energies and transfer integrals) described above. This can be done, e.g., with a transfer matrix approach [23].

#### 2.3.4. TB Parameters for a Wire Model Description

The TB parameters for a wire model description of charge transfer or transport can be obtained as follows. The transfer integral between monomers │λ〉 and $│\lambda^{\prime }\rangle$

(25) $$t_{\lambda ,\lambda^{\prime }}=\langle \lambda │{\hat{H}}_{DNA}│\lambda^{\prime }\rangle ,$$

can be analyzed as

(26) $$t_{\lambda ,\lambda^{\prime }}=\sum_{\nu =1}^{N_{\lambda }}\sum_{i=1}^{I_{\lambda }}\sum_{\mu =1}^{N_{\lambda^{\prime }}}\sum_{j=1}^{I_{\lambda^{\prime }}}c_{i\nu (\lambda )}^{*}\;V_{i\nu j\mu }\;c_{j\mu (\lambda^{\prime })},$$

where

(27) $$V_{i\nu j\mu }=\langle \phi_{i\nu (\lambda )}│{\hat{H}}_{\mathrm{DNA}}│\phi_{j\mu (\lambda^{\prime })}\rangle .$$

The matrix elements $V_{i\nu j\mu }$ are given by the Slater–Koster two-center interaction transfer integrals of Equations (7)–(8) with the values of $V_{\mathrm{ss}\sigma },V_{\mathrm{sp}\sigma },V_{\mathrm{pp}\sigma },V_{\mathrm{pp}\pi }$ being of the form of Equation (16). The tight-binding parameters $E_{\lambda }$ and $t_{\lambda ,\lambda^{\prime }}$ computed in this work could be used to treat charge transfer (Section 2.3.2) and transport (Section 2.3.3) along a B-DNA segment.

Finally, we obtain the maximum transfer percentage of the carrier from one base pair to another. This refers to the maximum probability to find the extra hole or electron at the site where it was not placed at initially. The maximum transfer percentage reads

(28) $$p=\frac{{\left(2t\right)}^{2}}{{\left(2t\right)}^{2}+\Delta^{2}}$$

where *t* is the transfer parameter between the two base pairs and Δ is the difference between the HOMO or LUMO energies of the two base pairs.

### 2.4. DNA Fragments Generated by MD

In order to study the effects of structural variability on the electronic structure and charge transfer parameters in B-DNA, we used multiple instances of AA and GG dimers. These instances were pruned from representative structures of the 500 ns MD trajectory of the 20mer $5^{\prime }-$CGAAAAGGGGAAAAGGGGAT$-3^{\prime }$ at constant temperature T=300 K and constant pressure P=1 bar. Specifically, we considered the centroid structures of the two most populated clusters, accounting for 35% (cl1) and 12% (cl2) of the whole trajectory. More details are given elsewhere [16]. From the two most representative 20mers we extracted all the possible AA and GG dimers (two stacked H-bonded base pairs), excluding the dimers of the edges. These dimers are denoted as: A4A5_cl1, A4A5_cl2, A5A6_cl1, A5A6_cl2, G7G8_cl1, G7G8_cl2, G8G9_cl1, G8G9_cl2, G9G10_cl1, G9G10_cl2, A11A12_cl1, A11A12_cl2, A12A13_cl1, A12A13_cl2, A13A14_cl1, A13A14_cl2, G15G16_cl1, G15G16_cl2, G16G17_cl1, G16G17_cl2. We denote the corresponding monomers as: A4_cl1, A4_cl2, A5_cl1, A5_cl2, A6_cl1, A6_cl2, G7_cl1, G7_cl2, G8_cl1, G8_cl2, G9_cl1, G9_cl2, G10_cl1, G10_cl2, A11_cl1, A11_cl2, A12_cl1, A12_cl2, A13_cl1, A13_cl2, A14_cl1, A14_cl2, G15_cl1, G15_cl2, G16_cl1, G16_cl2, G17_cl1, G17_cl2.

**Figure 1:**
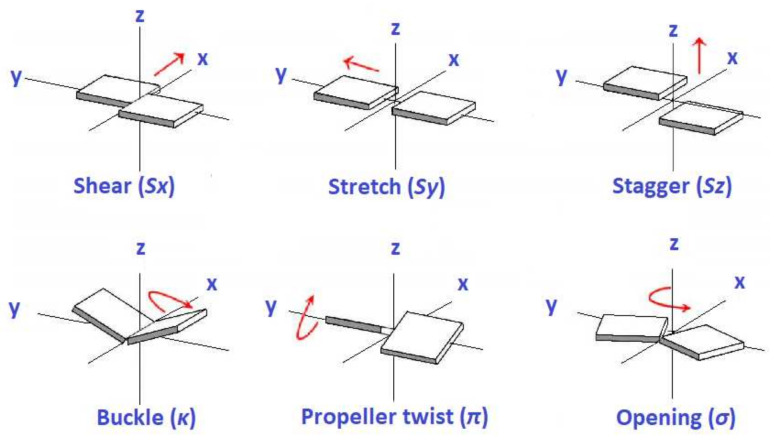
Definitions of translation parameters (**top row**) and rotation parameters (**bottom row**) involving two bases of a base pair.

Local complementary base-pair parameters are employed in order to define the base pair structure and its variability. The parameters describing the relative translations in all axes, involving two bases of a Watson–Crick pair, are shear (Sx), stretch (Sy), and stagger (Sz), while the corresponding rotations around *x*, *y*, and *z* axes are buckle (κ), propeller twist (π), and opening (σ) [24]. Figure 1 depicts the definitions of these translation and rotation parameters involving two bases of a Watson–Crick pair.

**Figure 2:**
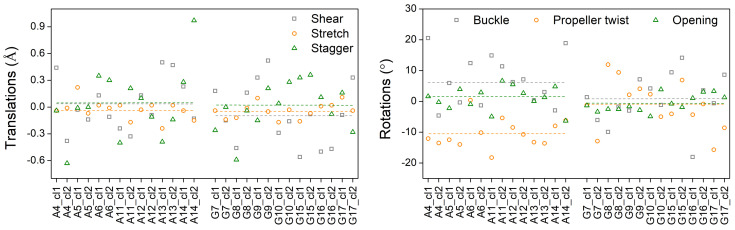
Translation (shear, stretch, stagger) and rotation (buckle, propeller twist, opening) parameters for all studied monomers. Dashed lines denote the mean value of each parameter.

Figure 2 sketches the translation and rotation parameters for each one of the studied monomers. The parameters were computed using the web interface 3DNA. Dashed lines denote the mean value of each parameter, that is: 0.03 Å (shear), −0.03 Å (stretch), 0.04 Å (stagger), $6.53^{\circ }$ (buckle), $-10.40^{\circ }$ (propeller twist), $1.06^{\circ }$ (opening) for A-T monomers and −0.09 Å (shear), −0.04 Å (stretch), 0.01 Å (stagger), $0.55^{\circ }$ (buckle), $-1.13^{\circ }$ (propeller twist), $-0.66^{\circ }$ (opening) for G-C monomers. These values together with values found in the literature are listed in Table 3.

## 3. Results and Discussion

### 3.1. Heterocyclic Planar Molecules including Nucleic Acid Bases

The theoretical scheme described in Section 2 was employed to calculate the HOMO and LUMO eigenenergies for a variety of heterocyclic planar organic molecules. We make the convenient simplifying assumption that the HOMO absolute value expresses the ionization energy, and the HOMO–LUMO gap expresses the excitation energy (in most cases the first π-$\pi^{*}$ transition). Below, the ionization energies are of π molecular orbital character and the excitation energies are π-$\pi^{*}$ transitions, unless otherwise stated. We studied the following groups of molecules: adenine and isomers; guanine and isomers; purine and isomers; thymine, cytosine, uracil, and isomers; pyrimidine and isomers; and other planar heterocyclic molecules. Table 4 summarizes our LCAO results using all valence orbitals, along with relevant experimental values. $I_{\mathrm{CC}}$ and $E_{\mathrm{CC}}$ are calculations of the vertical ionization energies at the Ionization Potential Equation of Motion Coupled Cluster with Singles and Doubles (IP-EOMCCSD)/aug-cc-pVDZ level of theory and vertical excitation energies at the Completely Renormalised Equation of Motion Coupled Cluster with Singles, Doubles, and non-iterative Triples (CR-EOMCCSD(T))/aug-cc-pVDZ level of theory, respectively, ref. [29].

Table 4 also includes transition oscillator strengths *f* that we calculated in a simplistic approximation, considering point contribution of the corresponding orbitals; i.e., the transition dipole moment $\vec{d}$ was approximated as

(29) $$\begin{array}{cc} \vec{d} & =\left(-e\right)\mathrm{\langle L│}\vec{r}│\text{H}\rangle =\left(-e\right)\left(\sum_{\nu =1}^{N}\sum_{i=1}^{I}c_{i\nu L}^{*}{\langle \phi }_{i\nu }│\right)\vec{r}\left(\sum_{\mu =1}^{N}\sum_{j=1}^{I}c_{j\mu H}{│\phi }_{j\mu }\rangle \right)\\ \; & =\left(-e\right)\sum_{\nu =1}^{N}\sum_{i=1}^{I}\sum_{\mu =1}^{N}\sum_{j=1}^{I}c_{i\nu L}^{*}c_{j\mu H}{\mathrm{\langle \phi}}_{i\nu }│\vec{r}│\phi_{j\mu \rangle }≃\left(-e\right)\sum_{\nu =1}^{N}\sum_{i=1}^{I}c_{i\nu L}^{*}\vec{r_{i}}c_{i\nu H}, \end{array}$$

where |*L*〉 (|*H*〉) is the LUMO (HOMO) state. The oscillator strength is [30]

(30) $$f=\frac{2}{3}\frac{m}{e^{2}\hbar^{2}}E\;d^{2}.$$

**Figure 3:**
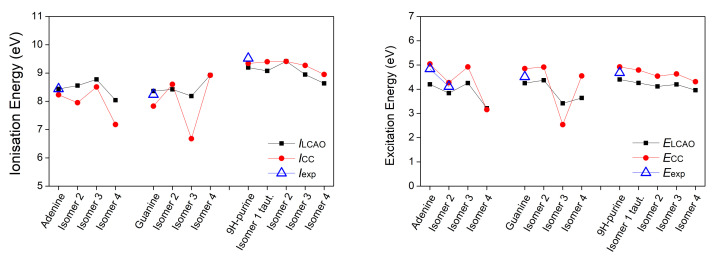
First π ionization energy and first π-$\pi^{*}$ excitation energy of purines calculated via our LCAO method using all valence orbitals, along with results at the IP-EOMCCSD/aug-cc-pVDZ (vertical ionization energies) and CR-EOMCCSD(T)/aug-cc-pVDZ (vertical excitation energies) level of theory [29], as well as available experimental data. Different isomers are specified in Table 1.

**Figure 4:**
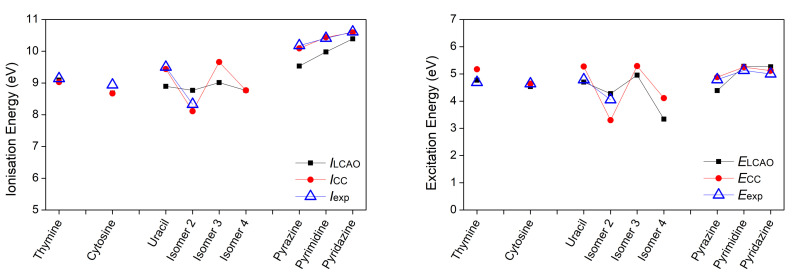
First π ionization energy and first π-$\pi^{*}$ excitation energy of pyrimidines calculated via our LCAO method using all valence orbitals, along with results at the IP-EOMCCSD/aug-cc-pVDZ (vertical ionization energies) and CR-EOMCCSD(T)/aug-cc-pVDZ (vertical excitation energies) level of theory [29], as well as available experimental data.

**Figure 5:**
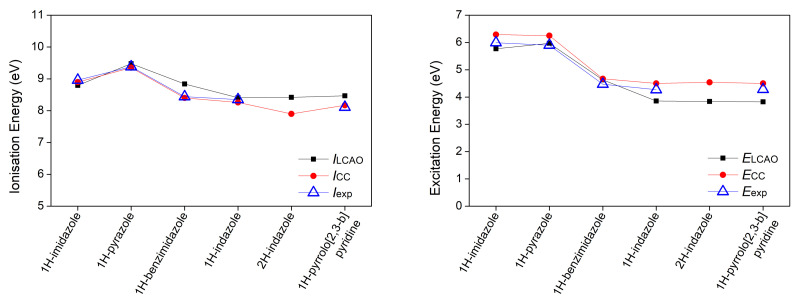
First π ionization energy and first π-$\pi^{*}$ excitation energy of other planar heterocyclic molecules calculated via our LCAO method using all valence orbitals, along with results calculated at the IP-EOMCCSD/aug-cc-pVDZ (vertical ionization energies) and CR-EOMCCSD(T)/aug-cc-pVDZ (vertical excitation energies) level of theory [29], as well as available experimental data.

*E* is the excitation energy. The results are illustrated in Figure 3, Figure 4 and Figure 5.

Regarding the ionization energy, the LCAO obtained results are in very good agreement with both the experimental data and the CC results, although there are some deviations. The Root Mean Square Percentage Error (RMSPE), with respect to the experimental values, is 3.65%. Differences in tautomer ionization energies are as expected negligible, that is 0.12 eV for purine tautomers and 0.01 eV for indazole tautomers. As for the excitation energies of the π-$\pi^{*}$ transition, the RMSPE, with respect to the experimental values, is 6.49%. Both purine and indazole tautomers have a negligible 0.03 eV difference in their excitation energies. Based on the presented data and reported comments about individual bases, we note that the LCAO method used in this work, though not exact, is capable of producing results in a good agreement with experimental data, when choosing the suitable set of parameters. This outcome has motivated the use of the same method for all other systems of interest, whose computational results are presented in the remainder of this article. Vertical ionization energies of nucleic acid bases in the gas phase with different electronic structure methods are, generally, in agreement with our results, cf. Reference [51] and references therein.

### 3.2. B-DNA Base Pairs

In this subsection, we present our results for the B-DNA base pairs. In Table 5, we show the HOMO, LUMO, and HOMO–LUMO gap energies of the two B-DNA base pairs (Adenine (A)-Thymine (T) and Guanine (G)-Cytosine (C)), according to the procedure described in Section 2.3 using LCAO with all valence orbitals, along with the corresponding energies found in Ref. [52] using only 2p${}_{z}$ orbitals. At this point, we should state that the bases making up the base pairs are slightly deformed in comparison to their structure when isolated (cf. Section 3.1), so the corresponding HOMO and LUMO energies for these two cases may differ. Thus, Table 5 also contains the HOMO, LUMO, and HOMO–LUMO gap energies of the distorted bases. The HOMO (LUMO) energies are of π ($\pi^{*}$) molecular orbital character and the HOMO–LUMO gap energies are π-$\pi^{*}$ transitions, unless otherwise stated.

The energy values for the bases are slightly different from those in Table 4, as expected. In addition, based on Table 5, one can assume that the HOMO energy of a particular base pair is very close to the largest of the HOMO energies of the two bases of the base pair, while the LUMO energy of the base pair is closer to the lowest of the two LUMO energies.

**Figure 6:**
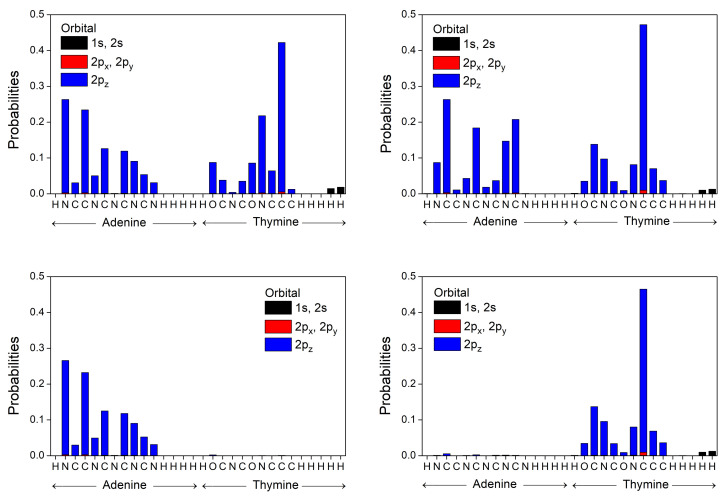
Occupation probabilities of each atomic orbital, $|c_{i\nu }{|}^{2}$ (cf. Equation (1)), for the HOMO **(left**) and LUMO (**right**) states of A and T bases into an A-T base pair (**top**), along with the corresponding probabilities (cf. Equation (12)) for the HOMO and LUMO states of the A-T base pair (**bottom**).

**Figure 7:**
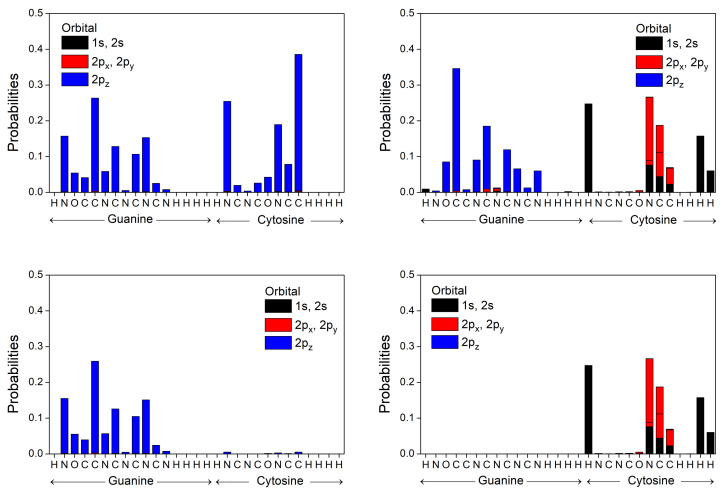
Occupation probabilities of each atomic orbital, $|c_{i\nu }{|}^{2}$ (cf. Equation (1)), for the HOMO (**left**) and LUMO (**right**) states of G and C bases into a G-C base pair (**top**), along with the corresponding probabilities (cf. Equation (12)) for the HOMO and LUMO states of the G-C base pair (**bottom**).

In Figure 6 and Figure 7 we represent the occupation probabilities of holes and electrons on each atomic orbital of bases and base pairs, calculating the squared coefficients $|c_{i\nu }{|}^{2}$ (cf. Equations (1) and (12)) of the corresponding states (HOMO for holes, LUMO for electrons). We observe that our calculated HOMO state for the base pair A-T (G-C) is localized almost totally in Adenine (Guanine), while the corresponding LUMO wave function is localized in Thymine (Cytosine), in accordance to results from ab initio techniques of References [53,54], which locate the HOMO of a base pair in purine and the LUMO in pyrimidine. This is due to the higher HOMO energy of Adenine (Guanine) and lower LUMO energy of Thymine (Cytosine) and the large values of these differences compared to the transfer integrals (see Table 6). We calculate the first transition character of A, T, A-T, and G to be π-$\pi^{*}$, while C and G-C have π-$\sigma^{*}$ transition character.

We obtain the charge transfer parameters between two successive base pairs by calculating the corresponding overlap integrals from Equation (26). We denote by XY two successive base pairs, X-X${}_{\mathrm{compl}}$ and Y-Y${}_{\mathrm{compl}}$. The bases X and Y are located at the same strand in the direction $5^{{}^{\prime }}$-$3^{{}^{\prime }}$, while X${}_{\mathrm{compl}}$ and Y${}_{\mathrm{compl}}$, respectively, are their complementary bases on the other strand. In the most common B-DNA conformation, X-X${}_{\mathrm{compl}}$ and Y-Y${}_{\mathrm{compl}}$ are approximately separated by 3.4 Å and twisted by 36${}^{\circ }$.

Table 6 summarizes our LCAO results using all valence orbitals for the transfer parameters, for all possible combinations of successive base pairs and close-to-ideal geometrical conformations. The Table also contains comparisons with other methods.

**Figure 8:**
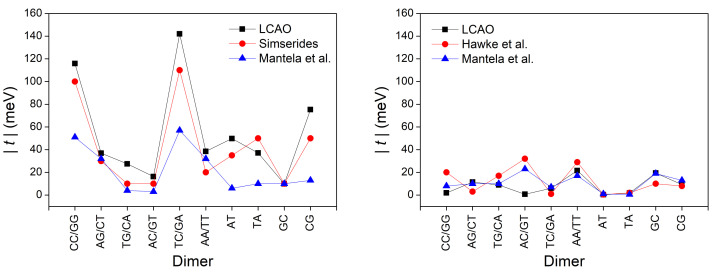
The absolute values of transfer parameters for all possible combinations of successive base pairs for holes (**left**) and for electrons (**right**). We show the transfer parameters obtained from our LCAO calculations using all valence orbitals, as well as the corresponding transfer parameters found in Reference [55] (for holes, estimation from various articles in bibliography), in Reference [29] (using only 2p${}_{z}$ orbitals) and in Reference [52] (for electrons, using only 2p${}_{z}$ orbitals).

In Figure 8, we illustrate the absolute values of transfer parameters for all possible combinations of successive base pairs for holes and for electrons. The figure contains the transfer parameters obtained from our LCAO calculations using all valence orbitals, along with the corresponding parameters found in Ref. [55] (where various estimations from bibliography had been taken into account). Furthermore, those from Ref. [29], where only 2p${}_{z}$ orbitals had been used, and finally, electron transfer parameters from Ref. [52], where only 2p${}_{z}$ orbitals had been used. Peluso et al. [56], based on electrochemical and time-dependent spectroscopic measurements, find for GG a transfer integral ≈ 0.1 eV, which is very close to our results, while, for AA, they report a value ≈ 0.3 eV, which seems large compared to the parametrization reported here taking into account all valence orbitals as well as to the parametrization in Reference [55], which takes into account, for holes, the works [52,57,58,59,60,61].

**Figure 9:**
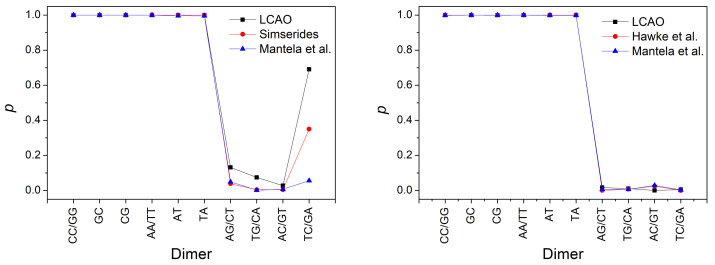
Comparison of the maximum transfer percentage *p* obtained by our LCAO method using all valence orbitals, with the *p* values extracted from other sources: obtained from parameters found in Reference [55] (for holes, estimation from various articles in bibliography), in Reference [29] (using only 2p${}_{z}$ orbitals) and in Reference [52] (for electrons, using only 2p${}_{z}$ orbitals). Left panel for holes, right panel for electrons.

In Figure 9, we depict the maximum transfer percentage of Equation (28) obtained by our LCAO calculations using all valence orbitals, compared to the values using parameters from Reference [55] for holes (an estimation from various articles from bibliography). Furthermore, from Reference [29] for electrons and holes as well as from Reference [52] for electrons (where only 2p${}_{z}$ orbitals had been used). For ideal B-DNA geometries and for dimers made of identical monomers, the maximum transfer percentage is 1, while in the case of different monomers, *p* is smaller than 1, both for holes and for electrons. Both for *t* and *p*, we observe that the current LCAO using all valence orbitals is closer to the results from Reference [55] for holes (where various estimations from bibliography of different origin had been taken into account). For electrons, as far as we know this current LCAO calculation is the only one beyond simple Hückel models, using only 2p${}_{z}$ orbitals.

### 3.3. Effects of Structural Variability

**Figure 10:**
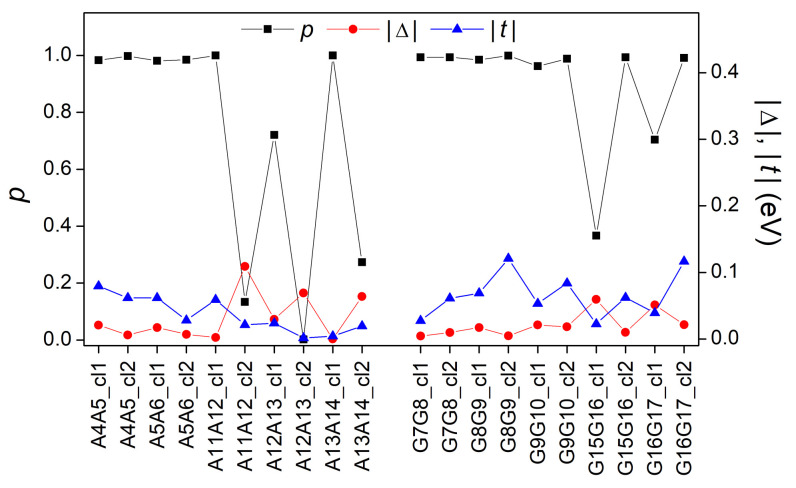
The parameters |Δ| and |t|, as well as the maximum transfer percentage *p* for all the dimers of the MD oligomer.

In this subsection, we analyze the effects of structural variability on the electronic structure and charge transfer properties of B-DNA using the fragments derived from MD, as detailed in Section 2.4. In Figure 10, we present the absolute values of the parameters Δ (difference between the HOMO eigenenergies of the two base pairs of each studied dimer) and *t* (transfer integral between the two base pairs’ HOMOs of each studied dimer), as well as the maximum transfer percentages *p* as calculated via Equation (28). The values of |t| and *p* can also be found in Reference [16] in comparison with results obtained by Density Functional Theory (DFT) techniques.

From Equation (28) it is expected that ideal dimers (made up of ideal monomers) should have a maximum transfer percentage equal to 1. However, by observing Figure 10, one can notice that not all AA and GG dimers have p=1. Specifically, dimers with a *p* considerably different from unit (and a Δ different than zero) are: A11A12_cl2, A12A13_cl1, A121A13_cl2, A13A14_cl2, G15G16_cl1, and G16G17_cl1. This is expected because the studied monomers are not ideal, which means their consisting bases have relative translations and rotations (Figure 1) as depicted in Figure 2. More specifically, a small *p* value is related to a large Δ value, in accordance with Equation (28). Thus, it is expected that the structural parameters (shear, stretch, stagger, buckle, propeller twist, opening) have a reasonable effect on the HOMO (and LUMO) base-pair energy values and consequently on the values of Δ and *p*. As for the contribution of transfer integrals *t* to the above discussion, it is documented in Reference [16].

## 4. Conclusions and Outlook

In this work, we computed the tight-binding parameters that are necessary for a wire-model description of longitudinal (axial) charge transfer through B-DNA. We took into account structural variability by carrying out these computations for multiple structures resulting from a classical trajectory.

We initially calculated the lowest ionization and excitation energies of various “ideal” (frozen) heterocyclic organic molecules with a biological function, including the DNA and RNA bases and isomers. We did so employing the LCAO approximation in a new parameterization that accounts for all valence orbitals, i.e., 2s, 2p${}_{x}$, 2p${}_{y}$, 2p${}_{z}$ orbitals for C, N and O atoms and 1s orbital for H atoms. This LCAO approach is more suitable than the standard LCAO parameterization to investigate non-planar geometries. We predict ionization and excitation energies with RMSPE 3.65% and 6.49%, respectively, compared to the experimental values. Based on these errors, we infer that the proposed computational strategy is an adequate tool for a quick and relatively accurate estimation of the electronic structure for a variety of organic molecules.

Using the computed energies of the HOMO and LUMO within the proposed LCAO method, we then evaluated the energy levels of DNA base pairs (A-T, G-C) and the transfer integrals between stacked base pairs. Our results are in good agreement with reference data. The obtained transfer integrals can be used in further studies of charge transfer/transport in DNA oligomers and polymers.

Finally, we addressed the impact of structural flexibility (dynamics) on the electronic structure and charge transfer ability of B-DNA. To this end, we applied our LCAO method to 20 AA and GG dimers, extracted from representative structures in a classical MD trajectory of a 20mer evolved for 500 ns. For all these systems, we calculated the parameters Δ and *t*, as well as the maximum transfer percentage between the two monomers of a dimer *p*. We found that the values of Δ and *p* are significantly affected by geometrical changes. Nevertheless, in the vast majority of the studied dimers, the maximum transfer percentage is very close to unity.

We suggest that the proposed methodology can be used in a high-throughput manner to characterize dynamical effects on charge transfer in organic polymers constituted of heterocyclic building blocks.

Our cost-effective simple method is suitable for very fast computations of electronic structure and transfer integrals. It can greatly facilitate charge transfer and transport calculations in sequences of arbitrary geometry taken, e.g., by MD simulations, as far as purines, pyrimidines, and similar molecules are the constituents. Although we took only valence orbitals for carbon, nitrogen, oxygen, and hydrogen into account, this approach can be generalized to include other atomic species and orbitals.

## Figures and Tables

**Table 1:** Diagonal matrix elements also known as on-site energies, in our LCAO parameterization (eV).

$E_{H(1s)}$	$E_{C(2s)}$	$E_{C(2p)}$	$E_{N(2s)}$	$E_{N(2p)}$	$E_{O(2s)}$	$E_{O(2p)}$
−13.64	−13.18	−6.70	−14.51	−9.55	−15.03	−11.52

**Table 2:** χ values of Harrison-type expressions for nondiagonal matrix elements, utilizing Slater–Koster two-center interaction transfer integrals, and the correction factor for interactions involving H atoms, in our LCAO parameterization.

$\chi_{\mathrm{ss}\sigma }$	$\chi_{\mathrm{sp}\sigma }$	$\chi_{\mathrm{pp}\pi }$	$\chi_{\mathrm{pp}\sigma }$	*b*
−1.32	−1.42	−0.73	2.22	0.70

**Table 3:** The second and third column contain mean values of translation and rotation parameters for monomers A-T and G-C, as studied in the present work. Other columns list values from bibliography.

Parameter	A-T	G-C	[25]	[26]	[27]	[28]
shear (Å)	0.03	−0.09	0.00	−0.04		
stretch (Å)	−0.03	−0.04	−0.15	−0.17		
stagger (Å)	0.04	0.01	0.09	0.21		
buckle (${}^{\circ }$)	6.53	0.55	0.5	0.3	(−7.5,7.5)	
propeller twist (${}^{\circ }$)	−10.40	−1.13	−11.4	−13.7	11.5	−12.60±3.2
opening (${}^{\circ }$)	1.06	−0.66	0.6	1.0	(−2,2)	

**Table 4:** Ionization and excitation energies (eV). $I_{\mathrm{LCAO}}$ and $E_{\mathrm{LCAO}}$ are the ionization and excitation energies obtained by our LCAO scheme, including all valence orbitals. $f_{\mathrm{LCAO}}$ is the relevant oscillator strength. $I_{\mathrm{CC}}$ and $E_{\mathrm{CC}}$ are the energies calculated at the IP-EOMCCSD/aug-cc-pVDZ and CR-EOMCCSD(T)/aug-cc-pVDZ level of theory [29]. $I_{\exp}$ and $E_{\exp}$ are the experimental data. In parentheses, the character of the transition.

Name Formula	$I_{\mathrm{LCAO}}$	$E_{\mathrm{LCAO}}$	$f_{\mathrm{LCAO}}$	$I_{\mathrm{CC}}$	$E_{\mathrm{CC}}$	$I_{\exp}$	$E_{\exp}$
Adenine							
$C_{5}H_{5}N_{5}$	8.44	4.20	0.330	8.23	5.04	8.44 [31]	4.84 [32,33]
*(Isomer 1)*							
2-Aminopurine							
$C_{5}H_{5}N_{5}$	8.56	3.84	0.239	7.95	4.27		4.11 [34]
*(Isomer 2)*							
1H-pyrazolo[3,4-d]							
pyrimidin-4-amine							
$C_{5}H_{5}N_{5}$	8.78	4.25	0.328	8.51	4.92		
*(Isomer 3)*							
Pyrimido [5,4-e]-as-							
triazine, 1,2-dihydro-							
$C_{5}H_{5}N_{5}$	8.04	3.21	0.282	7.18	3.16		
*(Isomer 4)*							
Guanine					4.77 ($\pi \to \sigma^{*}$)		
$C_{5}H_{5}N_{5}O$	8.36	4.25	0.288	7.83	4.85	8.24 [31]	4.51 [33]
*(Isomer 1)*							
7-Amino-S-triazolo(1,5-a)							
pyrimidin-5(4H)-one							
$C_{5}H_{5}N_{5}O$	8.42	4.37	0.285	8.60	4.91		
*(Isomer 2)*							
Pyrimido[5,4-e]-as-triazin-							
5[6h]-one, 1,2-dihydro-							
$C_{5}H_{5}N_{5}O$	8.19	3.42	0.198	6.68	2.54		
*(Isomer 3)*							
7H-imidazo[4,5-d]-v							
triazin-4-one, 6-methyl-					4.47 ($n\to \sigma^{*}$)		
$C_{5}H_{5}N_{5}O$	8.93	3.64	0.302	8.92	4.55		
*(Isomer 4)*							
9H-purine					4.49 ($n\to \pi^{*}$)		4.28 [35] ($n\to \pi^{*}$)
$C_{5}H_{4}N_{4}$	9.20	4.40	0.313	9.34	4.92	9.52 [31]	4.68 [35]
*(Isomer 1)*							
7H-purine				9.34 (*n*)	4.36 ($n\to \pi^{*}$)		
$C_{5}H_{4}N_{4}$	9.08	4.26	0.295	9.40	4.79		
*(Isomer 1 taut.)*							
1H-1,2,3-triazolo							
[4,5-b]pyridine					4.49		
$C_{5}H_{4}N_{4}$	9.42	4.12	0.340	9.41	4.54		
*(Isomer 2)*							
[1,2,4]Triazolo							
[1,5-a]pyrazine							
$C_{5}H_{4}N_{4}$	8.95	4.20	0.230	9.27	4.63		
*(Isomer 3)*							
[1,2,3]Triazolo							
[1,5-a]pyrazine							
$C_{5}H_{4}N_{4}$	8.64	3.96	0.172	8.95	4.31		
*(Isomer 4)*							
**Formula**	$I_{\mathrm{LCAO}}$	$E_{\mathrm{LCAO}}$	$f_{\mathrm{LCAO}}$	$I_{\mathrm{CC}}$	$E_{\mathrm{CC}}$	$I_{\exp}$	$E_{\exp}$
Thymine					5.07 ($n\to \pi^{*}$)		
$C_{5}H_{6}N_{2}O_{2}$	9.09	4.77	0.316	9.03	5.17	9.14 [31]	4.69 [33]
Cytosine							
$C_{4}H_{5}N_{3}O$	8.68	4.54	0.306	8.67	4.64	8.94 [31]	4.64 [33]
Uracil					5.03 ($n\to \pi^{*}$)		
$C_{4}H_{4}N_{2}O_{2}$	8.89	4.70	0.286	9.44	5.27	9.50 [31]	4.79 [33,35]
*(Isomer 1)*							
Pyrazine, 1,4-dioxide							
$C_{4}H_{4}N_{2}O_{2}$	8.77	4.28	0.403	8.11	3.30	8.33 [36]	4.05 [37]
*(Isomer 2)*							
4(1H)-pyrimidinone,							
6-hydroxy-							
$C_{4}H_{4}N_{2}O_{2}$	9.01	4.95	0.103	9.66	5.29		
*(Isomer 3)*							
Maleic hydrazide							
$C_{4}H_{4}N_{2}{)}_{2}$	8.77	3.34	0.113	8.77	4.11		
*(Isomer 4)*							
Pyrazine				9.49 (*n*)	4.07 ($n\to \pi^{*}$)	9.63 [38]	4.20 [39]
$C_{4}H_{4}N_{2}$	9.53	4.39	0.258	10.09	4.88	10.18 [38]	4.79 [40,41]
*(Isomer 1)*							
Pyrimidine					4.41 ($n\to \pi^{*}$)		4.35 [39]
$C_{4}H_{4}N_{2}$				9.56 (*n*)	4.84 ($n\to \pi^{*}$)	9.73 [38]	4.62 [40]
*(Isomer 2)*	9.98	5.28	0.249	10.44	5.25	10.41 [38]	5.13 [33,35,40,41]
Pyridazine					3.76 ($n\to \pi^{*}$)		3.70 [39]
$C_{4}H_{4}N_{2}$	9.41 (*n*)	4.28	0.000 ($n\to \pi^{*}$)	9.07 (*n*)	4.47 ($n\to \pi^{*}$)	9.31 [38]	
*(Isomer 3)*	10.39	5.26	0.253	10.59	5.12	10.61 [38]	5.00 [41]
1H-imidazole		4.97	0.000 ($\pi \to \sigma^{*}$)		5.50 ($\pi \to \sigma^{*}$)		
$C_{3}H_{4}N_{2}$	8.80	5.77	0.171	8.90	6.29	8.96 [42]	5.99 [43]
*(Isomer 1)*							
1H-pyrazole		5.69	0.000 ($\pi \to \sigma^{*}$)				
$C_{3}H_{4}N_{2}$	9.69	5.90	0.000 ($\pi \to \sigma^{*}$)		6.11 ($\pi \to \sigma^{*}$)		
*(Isomer 2)*	9.48	5.97	0.196	9.35	6.25	9.38 [44]	5.90 [45]
1H-benzimidazole							
$C_{7}H_{6}N_{2}$	8.84	4.63	0.245	8.40	4.67	8.44 [42]	4.47 [46]
*(Isomer 1)*							
1H-indazole							
$C_{7}H_{6}N_{2}$	8.41	3.85	0.217	8.26	4.50	8.35 [47]	4.27 [48]
*(Isomer 2)*							
2H-indazole							
$C_{7}H_{6}N_{2}$	8.42	3.84	0.229	7.90	4.54		
*(Isomer 2 taut.)*							
1H-pyrrolo[2,3-b]							
pyridine							
$C_{7}H_{6}N_{2}$	8.47	3.82	0.184	8.17	4.50	8.11 [49]	4.28 [50]
*(Isomer 3)*							

**Table 5:** HOMO ($E_{\mathrm{LCAO},H}$) and LUMO ($E_{\mathrm{LCAO},L}$) eigenenergies of the base pairs A-T and G-C, obtained in this work using LCAO with all valence orbitals, along with the corresponding HOMO–LUMO energy gaps ($E_{\mathrm{LCAO},g}$) in eV (rows 6 and 7). Rows 2–5 contain the calculated HOMO and LUMO energies of each distorted base making up these base pairs. The third, fifth, and the seventh columns list the corresponding energies from Reference [52] where only 2p${}_{z}$ orbitals had been used.

Base or Base Pair	$E_{\mathrm{LCAO},H}$	$E_{H}$ [52]	$E_{\mathrm{LCAO},L}$	$E_{L}$ [52]	$E_{\mathrm{LCAO},g}$	$E_{g}$ [52]
A	−8.50	−8.30	−4.19	−4.40	4.31	3.90
T	−9.12	−9.00	−4.30	−4.90	4.82	4.10
G	−8.31	−8.00	−4.12	−4.50	4.19	3.50
			−4.43 ($\sigma^{*}$)		4.24 ($\pi \to \sigma^{*}$)	
C	−8.67	−8.80	−4.11	−4.30	4.56	4.50
A-T	−8.49	−8.30	−4.31	−4.90	4.18	3.40
			−4.43 ($\sigma^{*}$)		3.87 ($\pi \to \sigma^{*}$)	
G-C	−8.30	−8.00	−4.14	−4.50	4.16	3.50

**Table 6:** Close-to-ideal geometrical conformations. The absolute values of transfer parameters for all possible combinations of successive base pairs. $\left|t_{\mathrm{LCAO},H}\right|$ ($\left|t_{\mathrm{LCAO},L}\right|$) of the second (fifth) column refer to hole (electron) transfer parameters obtained from our LCAO calculations using all valence orbitals. The third column lists hole transfer parameters of Reference [55], an estimation from various articles found in bibliography. The sixth column lists the electron transfer parameters of Reference [52], where only 2p${}_{z}$ orbitals had been used. The fourth and seventh columns list the transfer parameters with the parameterization of Reference [29], where only 2p${}_{z}$ orbitals had been used. All transfer parameters are given in meV.

XY	$\left|t_{\mathrm{LCAO},H}\right|$	$\left|t_{H}\right|$ [55]	$\left|t_{H}\right|$ [29]	$\left|t_{\mathrm{LCAO},L}\right|$	$\left|t_{L}\right|$ [55]	$\left|t_{L}\right|$ [29]
				92 ($\sigma^{*}$)		
GG, CC	116	100	51	2	20	8
				11 ($\sigma^{*}$)		
AG, CT	37	30	32	11	3	10
				2 ($\sigma^{*}$)		
TG, CA	28	10	4	9	17	10
				1 ($\sigma^{*}$)		
AC, GT	16	10	3	1	32	23
				3 ($\sigma^{*}$)		
TC, GA	142	110	57	6	1	7
AA, TT	38	20	32	22	29	17
AT	50	35	6	1	1	1
TA	37	50	10	2	2	1
				2 ($\sigma^{*}$)		
GC	10	10	10	19	10	19
				1 ($\sigma^{*}$)		
CG	75	50	13	9	8	13

## Data Availability

Data available upon reasonable request.

## Abbreviations

LCAO	Linear Combination of Atomic Orbitals
MD	Molecular Dynamics
DNA	Deoxyribonucleic Acid
TB	Tight Binding
IP-EOMCCSD	Ionization Potential Equation of Motion
	Coupled Cluster with Singles and Doubles
CR-EOMCCSD(T)	Completely Renormalized Equation Of Motion
	Coupled Cluster with Singles, Doubles, and non-Iterative Triples
RMSPE	Root Mean Square Percentage Error
HOMO	Highest Occupied Molecular Orbital
LUMO	Lowest Unoccupied Molecular Orbital
